# ‘It is important to feel invited’: what patients require when using the Utrecht Symptom Diary – 4 Dimensional, a qualitative exploration

**DOI:** 10.1177/26323524241260426

**Published:** 2024-06-20

**Authors:** Tom Lormans, Everlien de Graaf, Sita de Vries, Carlo Leget, Saskia Teunissen

**Affiliations:** Julius Center for Health Sciences and Primary Care, University Medical Center Utrecht, Universiteitsweg 100, Utrecht 3584CG, The Netherlands; Julius Center for Health Sciences and Primary Care, University Medical Center Utrecht, Utrecht, The Netherlands; Julius Center for Health Sciences and Primary Care, University Medical Center Utrecht, Utrecht, The Netherlands; Care Ethics, University of Humanistic Studies, Utrecht, The Netherlands; Julius Center for Health Sciences and Primary Care, University Medical Center Utrecht, Utrecht, The Netherlands

**Keywords:** autonomy, communication, palliative care, patient-reported outcome measures, sociality, spirituality

## Abstract

**Background:**

In palliative care, the Utrecht Symptom Diary – 4 Dimensional (USD-4D), a Dutch-adapted and validated patient-reported outcome measure, supports multidimensional symptom management through identification and monitoring of, as well as dialogue on symptoms and needs. For the USD-4D to optimally support patients’ autonomy, it is essential to know what patients need to use it.

**Objective:**

This study aims to identify what patients need when using the USD-4D in clinical palliative care.

**Design:**

A generic qualitative design with primary and secondary analyses of semistructured interviews.

**Methods:**

Patients ⩾18 years with a life-limiting illness were purposefully recruited within hospice and home care settings if they were in their last year of life as identified by the surprise question. Patients had to be aware of their life-threatening condition. Patients were selected in two tranches. In the first tranche, patients had to have completed the USD-4D at least once. The second tranche consisted of patients who were not familiar with the USD-4D in clinical practice and were interviewed in a previous study on the content validity of the USD-4D. The interviews were transcribed verbatim and were subjected to thematic analysis.

**Results:**

Twenty-five patients were included (14 men, ages 44–87). Patients’ needs when using the USD-4D were summarized in three themes: (1) feeling invited, (2) being aware of the purpose and function of the USD-4D, and (3) experiencing a personal and nonjudgmental approach.

**Conclusion:**

For patients to optimally benefit from the USD-4D as a supportive measure of their autonomy in clinical palliative care, it is essential that they feel invited to use it. Healthcare providers are tasked with setting the right preconditions for patients to want and to be able to use the USD-4D. For patients, this means healthcare providers should always be attuned to their personal preferences when communicating the purpose and function of the USD-4D and when they enter into dialogue with them.

## Introduction

Patients with life-limiting illnesses experience multidimensional symptoms and needs that require personalized care.^1^ The World Health Organization stresses the importance of assessing, exploring and discussing physical, psychological, social and existential symptoms and needs to optimize patients’ quality of life.^2^ The use of patient-reported outcome measures (PROMs) is recommended to support both patients and healthcare providers (HCPs) in identifying, monitoring, and discussing patients’ symptoms, needs, and wishes, thus supporting autonomy and patient-centered care.^3–6^

Internationally, the Edmonton Symptom Assessment System (ESAS) has proven to be a feasible instrument in symptom assessment and monitoring in clinical care.^7,8^ The Utrecht Symptom Diary (USD) is a Dutch-adapted version of the ESAS and focuses on common physical and psychological symptoms in palliative care patients with items added for well-being and value of life.^9^ The USD was designed for patients to self-assess their multidimensional symptoms and needs as an integrated part of routine care. The Netherlands Quality Framework for Palliative Care recommends the use of the USD in palliative care.^10^

Four-dimensional PROMs have not yet been developed for use in clinical palliative care. Within clinical palliative care as well as various working groups organized in Dutch palliative care networks, HCPs have indicated that the development of a multidimensional PROM would support personalized multidimensional palliative care in daily practice. A first step has been taken with the development of the Utrecht Symptom Diary – 4 Dimensional (USD-4D) (Supplemental Appendix 1). To cover all four dimensions, five expert-based items that concern the social and spiritual dimensions were added to the original USD. Including the addition of these five items, the USD-4D contains a total of 21 items that are assessed using an 11-point numerical intensity scale (0 = no symptom, best possible; 10 = worst intensity, worst possible). Moreover, patients are encouraged to prioritize their symptoms and needs in a summarizing question.

These five items were derived from a spiritual care instrument frequently used in Dutch palliative care: the Diamond Model, which is an operationalization of the Ars Moriendi tradition.^11^ The Diamond Model is a validated hermeneutic instrument that is used to support patients and HCPs with conversations on social and spiritual issues in Dutch palliative care.^12^ The Diamond Model is feasible in clinical palliative care for exploring and interpreting patients’ social and spiritual needs and entering into dialogue on them.^13^ The theoretical foundation of the Diamond Model has been translated into English, German, and Spanish.^11^

**Figure 1.**
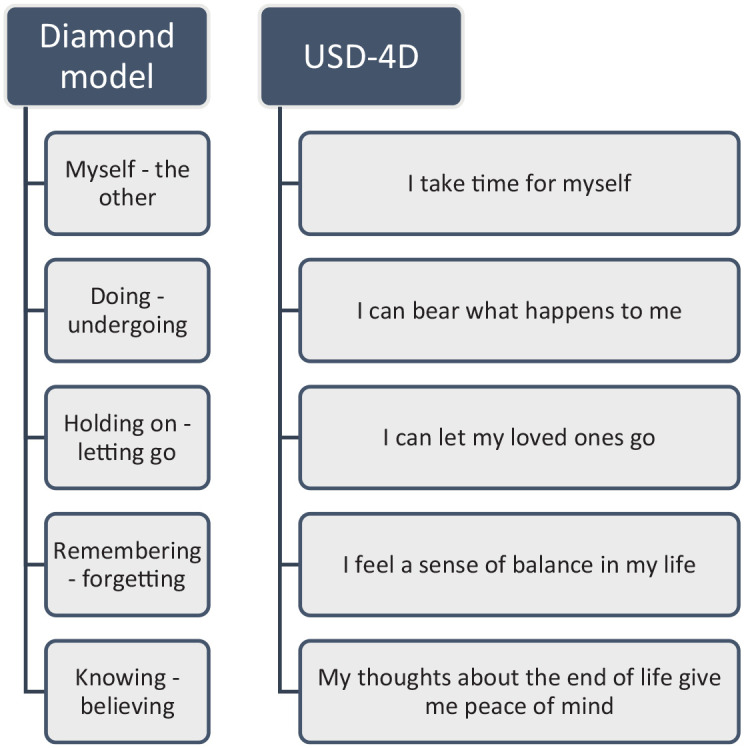
Anthropological polarities of the Diamond Model^11^ and their respective operationalized USD-4D items. USD-4D, Utrecht Symptom Diary – 4 Dimensional.

Central to the Diamond Model is the concept of inner space. Inner space is a metaphor that refers to the state of mind that allows one to relate to immediate emotions and attitudes evoked by a situation. Five polarities support exploring whether someone can experience inner space: myself *versus* the other, doing *versus* undergoing, holding on *versus* letting go, remembering *versus* forgetting, and knowing *versus* believing.^11^ For the USD-4D, the five polarities of the Diamond Model have been put into operation as five sociospiritual items by clinical palliative care experts: healthcare chaplains, psychologists, and palliative care nurses − depicted in Figure 1. In the development process of the USD-4D, chaplains, nurses, and patients were consulted to give feedback on all versions prior to the final version that is a part of this study. Also, patients were directly involved in the validation of the USD-4D as part of the content validity study of the social and spiritual items.^14^ Other validation studies have been carried out.

Using the USD-4D involves patients completing the USD-4D and entering into dialogue with HCPs to explore and/or understand underlying wishes and care needs. Generally, nurses invite patients to complete the USD-4D digitally using a tablet. Sometimes patients fill in the items on a printout version.^3,15^ A recent systematic review found that patients express their needs considering the social and spiritual dimensions in a similar fashion. Depending on the patients’ point of view, the items reflect either the social or spiritual dimension or both.^16^ In another study, patients were purposefully recruited to reflect on the content validity of the USD-4D items related to the social and spiritual dimensions and declared that these items were comprehensible, relevant, and comprehensive. These items enabled patients to express their issues and needs when considering these aspects of their life in light of their end-of-life problems.^14^

PROMs are being used in routine care and appear to help in identifying patients’ needs and, among other things, improve HCP-patient communication.^17^ Overall, PROMs support and improve the palliative care process.^18^ However, there are also challenges regarding the feasibility of PROMs, their benefits and the patients that ought to use them.^19,20^ A recent systematic review states that implementing a PROM asks for long-term strategy that is unlikely to succeed if the staff is not convinced of its benefits and feasibility.^18^ However, patients themselves should also perceive PROMs as beneficial.

Studies often focus on patients’ perception of the instrumental value of PROMs. On the one hand, patients perceive improved communication and increased awareness of issues through PROMs; on the other hand, they often see certain PROM items as irrelevant and do not have a clear understanding of PROMs.^21^ Patients are becoming increasingly involved in the development of PROMs and optimizing their comprehensibility.^22,23^ However, patients’ needs when using PROMs are taken less into account despite the fact that patients are actually expressing needs when using PROMs..^14,24–26^

Many challenges that affect a successful implementation and embedment of the USD-4D, and possibly more PROMs in general, can be overcome by addressing patients’ needs in using this PROM.^10^

It is essential to better understand what patients need to optimally engage with the USD-4D and to ensure that this leads to improved person-centered care provision.^25,26^ This study therefore aims to explore what patients need when using the USD-4D to enable the identification of their unique needs to inform tailored care planning and provision, specifically considering the items that concern the social and spiritual dimensions. By focusing on patients’ needs, this study fills a gap in knowledge about the implementation of PROMs.

## Methods

### Design

This qualitative exploratory study utilizes primary and secondary analyses to gain an in-depth understanding of patients’ needs when using the USD-4D. A primary study with a generic qualitative design was conducted from May 2019 to October 2020.^27^ Additionally, a secondary analysis was conducted on prior interviews to supplement the primary data. For the writing of this report, we adhered to the Standards for Reporting Qualitative Research.^28^

### Study objective

The objective of this study was to assess patients’ needs when using the USD-4D in clinical palliative care. These needs were operationalized as needs with respect to the patient’s self (e.g. calmness, time), the other (e.g. caregiver, attitude), location (e.g. quietness, privacy), and time (e.g. enough time, timing).^29^ In addition, patient demographics were collected: age, gender, marital status, level of education, illness, and religious or spiritual orientation.

### Population, setting, and sampling

The study population consisted of patients in the palliative phase of illness coming from two cohorts: in addition to the primary cohort (actual experienced needs, AN), sampled specifically for this research question, a second cohort (perceived needs, PN) that was used to study the content validity of the USD-4D was selected for its evident applicability to the present research question.^14^ For the interpretation of the results, this leads to the first cohort providing information on AN and the second cohort providing data on PN.

For both cohorts, eligible patients were ⩾18 years of age with an estimated life expectancy of ⩽1 year. The latter was assessed through a negative answer to the ‘surprise’ question: ‘Would I be surprised if this patient died in the next 12 months?’ When the surprise question was answered with ‘no’, patients were eligible.^30^ Furthermore, patients needed to be aware of their life-limiting disease. For the first cohort, patients had to have completed the USD-4D at least once, while in the second cohort, this was not required. In both cohorts, patients were excluded if they did not adequately understand the Dutch language or if they were not capable of being interviewed for 15–45 min. Moreover, Dutch citizens with a migration background were excluded to avoid high complexity in this explorative study.^31,32^ Purposeful sampling was performed to ensure maximum variation in age, gender, religion, and educational level over the two cohorts.^33^

### Study procedures

For the first cohort, one hospice and one home care organization in the center of the Netherlands were invited to participate in this study. Eligible patients were informed and invited for participation by a nurse practitioner. After patients gave consent, contact information was provided to the research team and the researcher (TL) contacted them with further information. Patients were given the opportunity to ask for more information before consenting to participate. If patients consented, the researcher contacted the patient to make an appointment for an interview. The interview was conducted at a location preferred by the patient and lasted 15–45 min. Before each interview, whether written or verbally recorded, informed consent was obtained.

For the second cohort, two home care services, nursing wards in one general hospital and a hospice, all in the center of the Netherlands, were invited to participate.^14^

### Data collection

Interviews were conducted by one researcher (TL) with a background in chaplaincy between November 2019 and December 2020. Training in qualitative research was received during initial education and expanded on during the study period through consultations with a senior researcher (EdG). The researcher had no relationship with the participants prior to study commencement.

Each interview started with a rapport building question: ‘How do you feel at this moment?’ Thereafter, the USD-4D was presented in print so that it could be always made clear which item was referred to. The data collection began with asking patients if they recalled using the USD-4D.

To increase dependability, a topic list (Supplemental Appendix 2) was employed to explore what patients need when using the USD-4D.^34^ Interviews were audio-recorded digitally and were stored on a secured hard drive. These recordings were then transcribed verbatim. The transcriptions were stored anonymously.^35,36^

In addition to these interviews, interviews from the second cohort were used. These interviews were conducted by a researcher (SdV) that also had no prior relationship to the patients. Patients were handed the USD-4D in print before the interviews commenced. From these interviews data were selected that described patients’ needs in using the USD-4D.

Data were collected until inductive thematic saturation was met through a code meaning approach.^37^ Analyses were conducted every three interviews. When a certain code was fully understood and no new aspects or nuances of this code were noted in subsequent interviews, this was an indicator of saturation. When a certain code was saturated, subsequent interviews focused less on this code and more on codes not yet saturated.^38^ The topic list would be adjusted to meet this end.

The way in which data were collected for the secondary analysis is described in de Vries *et al*.^14^

### Data analysis

**Figure 2.**
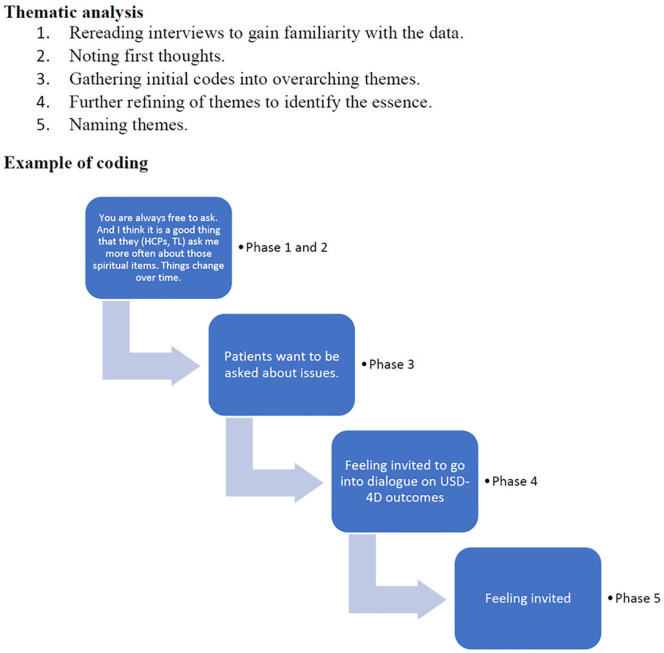
Process of thematic analysis and example of coding.

For the first cohort, interviews were discussed and analyzed iteratively using thematic analysis (see Figure 2). In collaboration with a senior researcher (EdG), themes were discussed, reworked, and refined to optimally fit the data. Continuous cycling between data and identified themes enabled optimal response to the research question.^39^ Furthermore, preliminary results were presented to a group of patients’ representatives (*n* = 2) and palliative care nurses (*n* = 2) to ensure that all key elements were explored. The final analysis also included the interviews conducted in the second cohort. NVivo 12 QSR International software was used to assist in the analysis.^40^ All analyses were conducted in Dutch. When extracts were selected for this article, they were translated in English by the research team (TL, EdG, CL, ST).

### Rigor

To optimize understandability and comprehensiveness, the topic list was assessed by a group of patient representatives (*n* = 2) and palliative care nurses (*n* = 2). Changes were made to the formulation of the proposed questions to ensure that they were easy for participants to understand. The topic list was completed within the research group (TL, EdG, CL, ST) (Supplemental Appendix 2). To increase the validity of the researcher’s interpretations, member checks were used during the interviews. By reformulating and mirroring participants’ statements, the interviewer checked to see if he understood the matter correctly. If this turned out not to be the case, further questions were asked in order to understand the participant.^41^ Two researchers (TL and EdG) coded the transcribed interviews and discussed these codes every three interviews to achieve a common understanding. Additionally, to ensure the correct interpretation of the results, they were presented to patient representatives (see above) and compared to the researchers’ interpretations. To document any contextual information that might be relevant for interpreting the results, field notes were written after each interview.^42^

### Ethical issues

The study was conducted in accordance with the principles of the Declaration of Helsinki^43^ and the General Data Protection Regulation.^44^ This research was not considered subject to the Medical Research Involving Human Subjects Act by the Medical Research Ethics Committee (MERC) Utrecht (19-182/C, March 2019). The MERC Utrecht also confirmed that the Medical Research Involving Human Subjects Act did not apply to the study concerning the content validity of the USD-4D that served the secondary analysis (19–053/C, January 2019).^14,45^ All participants provided verbal consent to be approached by the researcher.

## Results

In total, 25 interviews with patients with a life-limiting illness were analyzed. Thirteen patients were enrolled in this study and were interviewed about their needs using the USD-4D (AN). Twelve interviews concern secondary analyses of prior interviews that were analyzed for patients’ PN using the USD-4D (PN).

All in all, the 25 interviews included fourteen men (56%) with a mean age of 70 (range: 44–87 years). For 88% of the participants, the primary diagnosis was cancer. Thirteen participants identified themselves as Christians (52%), four of which were practicing (16%). Twelve patients were admitted to a hospice (48%). Table 1 shows the characteristics of all participants. Interviews were performed at a location preferred by the patient: either in their room in the hospice or hospital or at home. Interviews lasted between 21 and 67 min.

**Table 1.** Characteristics of the study population.

ID	Age	Sex	Marital status*	Care setting	Admission	Illness	Education**	Religion or ideology	Practicing religion or ideology
AN01	53	F	M	Hospice	Aug 2019	Cancer	High	Christianity	No
AN02	80	M	M	Hospice	Sept 2019	Cancer	High	None	No
AN03	75	F	W	Hospice	July 2019	Cancer	High	Christianity	No
AN04	67	F	M, Ch	Hospice	Oct 2019	Cancer	High	Christianity	Yes
AN05	79	F	W, Ch	Hospice	Nov 2019	Cancer	High	Humanism	No
AN06	78	M	M, Ch	Home	NA	Cancer	High	None	No
AN07	68	F	M	Home	NA	Cancer	High	None	No
AN08	77	M	M, Ch	Hospice	Jul 2020	Cancer	High	Christianity	No
AN09	62	M	M, Ch	Hospice	Jun 2020	COPD	High	None	No
AN10	67	M	M, Ch	Hospice	Jul 2020	Cancer	High	Christianity	No
AN11	44	M	M, Ch	Hospice	Aug 2020	Cancer	High	None	No
AN12	83	F	M	Hospice	Aug 2020	Cancer	High	Christianity	No
AN13	72	M	W	Hospice	Aug 2020	Cancer	High	Christianity	No
PN01	71	M	W, M	Home	2018	Cancer	Low	Christianity	Yes
PN02	62	M	M	Hospice	2018	ALS	Middle	Christianity	No
PN03	67	F	W	Home	2018	Cancer	Middle	None	NA
PN04	74	M	M	Home	2008	Cancer	Middle	None	NA
PN05	67	F	D	Home	2019	Cancer	High	None	NA
PN06	71	M	W, M	Home	2018	Cancer	High	Humanism	Yes
PN07	53	F	M	Home	2015	Cancer	Middle	Christianity	No
PN08	75	M	W	Hospital	2018	Cancer	Middle	Christianity	Yes
PN09	87	F	W	Home	2018	Cancer	High	Christianity	No
PN10	72	F	S	Home	N.A.	Cancer	Low	None	NA
PN11	62	M	S	Home	N.A.	Cancer	Low	None	NA
PN12	76	M	M	Hospital	2011	Cancer	Low	Christianity	No

* M: married/living together; Ch: children; W: widowed; D: divorced; S: single.

** Low: primary school, lower secondary general, lower vocational; middle: higher secondary general education, intermediate vocational education; high: higher vocational education, university.

NA: not applicable; ALS: amyotrophic lateral sclerosis; COPD: chronic obstructive pulmonary disease.

### Patients’ needs when using the USD-4D

Throughout the interviews, patients expressed personal and emotional preconditional needs in using the USD-4D in clinical practice. The needs can be summarized in three themes: (1) feeling invited, (2) being aware of the purpose and function of the USD-4D, and (3) experiencing a personal and nonjudgmental approach.

#### Theme 1: Feeling invited

Patients expressed the need to feel invited to use the USD-4D and enter into dialogue with HCPs about the meaning of their outcomes. Being invited gave them the opportunity to accept or decline the invitation. It also gave them the freedom to choose when and how they wanted to use the PROM. Furthermore, being invited did not feel burdensome to them because the patients themselves decided what they would do with it.

##### Accepting or declining the invitation

As one patient explained: ‘A nurse invited me in a very polite manner. She asked whether I wanted to use the USD-4D and did not tell me I should. I do not like people telling me what to do’ (AN09: male, 62). For patients, it was essential that they felt they had the opportunity to decline using the USD-4D. One patient (AN10: male, 67) told how the USD-4D items motivated him to enter into dialogue about underlying themes. Other patients stated that they did not want to go into depth about the USD-4D since it added no value for them (AN07: female, 68; PN10: female, 70). As one patient stated: ‘It doesn’t have any added value for me, so I answer the questions because it’s asked of me. But I don’t need to enter into dialogue about my answers in detail. And I think it is important that I can make that choice’ (AN08: male, 77). Patients appreciated that this was respected.

##### Freedom to choose

Being invited to use the USD-4D also involved patients themselves choosing a suitable time to do so: ‘It is up to me when I fill in the USD-4D and I appreciate that freedom’ (AN02: male, 80). Another patient stated: ‘The nurse sometimes says: “I’ll come back later.” And that’s fine for me. I don’t need to use the USD-4D right away, I can do it when I feel like doing it’ (AN03: female, 75).

Patients conveyed the importance of having a grip on their own disease process and being able to decide for themselves whether to use the USD-4D and enter into dialogue or not: ‘I have less need to talk to healthcare professionals. Sharing stories about, uhm, about my life is something I do when friends visit me, and I get all the space I need to do that. . . . So that is nice. Filling in the items is fine, but I do not wish to enter into dialogue on my answers. HCPs do not insist on going into dialogue’ (AN04: female, 67). Another patient said: ‘When they ask me to use the USD-4D, I do not feel I have to. I can say no if I do not want to, and I like having that freedom’ (AN12: female, 83).

##### Not burdened

All patients declared that they did not feel burdened by being invited to use the USD-4D. One patient said: ‘You are always free to ask. And I think it is a good thing that healthcare professionals ask me more often about those spiritual items. Things tend to change over time’ (PN10: female, 72). Moreover, when the patients felt that the social and spiritual items were more complex than the physical and psychological items, this did not have anything to do with the question in itself. Rather, it meant patients needed more time to think about the questions. Furthermore, patients stated that using the USD-4D was not demanding when properly introduced and embedded in their personal care process.

#### Theme 2: Being aware of the purpose and function of the USD-4D

Patients stated that it was important to them to feel that using the USD-4D was in some way useful to them. They did not want to have the feeling they were answering questions that were simply to gather information. Patients needed to perceive the purpose and function of the USD-4D and HCPs needed to explain this to them.

##### Sense of individual benefit

Patients stated it was important that they know using the USD-4D benefitted their personal care. A patient said: ‘Healthcare professionals should not only explain what the items entail, but also why I am actually answering the questions. For me, that is important in wanting to answer them’ (PN07: female, 53). Another patient stated: ‘If I did not know answering these questions would benefit my care, I would not like answering them’ (AN03: female, 75). Some patients stated that they were not aware of why the USD-4D was used: ‘Nurses ask me to answer these questions. But for what? That is not clear to me’ (AN10: male, 67). Another patient thought the USD-4D was to be used for scientific purposes: ‘I did mention right at the start I did not like the survey. . . . I know there is a lot of scientific research to be done. It’s all useful. And I understand, all those people who are studying something need to discover new things all the time. But I do not like all these questions’ (AN12: female, 83). One patient stated that it was pleasant the USD-4D did not contain many items (AN04: female, 67).

##### Use of and feedback on outcomes

Patients were more likely to use the USD-4D if they received feedback on their outcomes and were more involved in care processes involving the PROM: ‘I would be more willing to answer the questions if nurses told me what this means for the care I receive’ (PN10: female, 72). All patients expressed the fact that it was important to them that their outcomes were used in day-to-day care. One patient stated that he had experienced more than once that HCPs did not read up on his situation and that he had to tell his story again and again. After using the USD-4D, patients also wanted to be kept in the loop about what happened with their outcomes. As one patient clearly stated: ‘Healthcare professionals come back some time later and talk to you about the answers you’ve given. I would like to see even more. It would be nice, for instance, to see your past answers in a graph’ (AN11: male, 44). However, some patients explained that no dialogue was initiated by the HCP. This hindered them from wanting to use the instrument in further instances. One patient thought nothing was done with her answers: ‘I answer the questions when I feel like it and I don’t really see much of it. . . . Afterward nothing much happens with it in my opinion’ (AN06: female, 78).

##### Outcome scoring

USD-4D items are assessed using an 11-point numerical intensity scale (0 = no symptom, best possible; 10 = worst intensity, worst possible). For some patients, this was counter intuitive when it concerned the social and spiritual items. One patient expressed she did not pay attention to the instructions properly and simply missed how the items should be answered: ‘Well, I just did not pay attention . . . and I just assumed that a higher score means positive outcome, but it should have been the other way around’ (PN10: female, 72). Another patient stated that she had difficulties expressing herself in numbers and that she needed support from an HCP to do so (PN05: female, 67).

#### Theme 3: Experiencing a personal and nonjudgmental approach

Patients stressed the importance of a personal and nonjudgmental approach: being approached by HCPs in a way that supported their decision-making process. Moreover, they needed a trusting relationship with HCPs that allowed them to express themselves properly.

##### Attunement to personal context

Every interview showed that patients do not have a common understanding of the items and do not have any common knowledge on the underlying dimensions. Some patients think in abstract ideas, others in more tangible examples. This makes patients want HCPs to attune the questions to their personal context and level of knowledge. One patient said: ‘The first time they asked me about, what did they call it? The existential questions − it’s a difficult word. And then you think: existential? What kind of questions are those? They need some explanation. The people here have experience with other patients answering these questions, so they ought to know how to guide me towards the right answers’ (AN03: female, 75). Another patient said: ‘Nurses do not always have to explain the questions to me, I’m used to talking about my religion and how it affects my attitude towards death’ (PN05: female, 67). For some patients, a specific item might need more explanation or guidance than others.

Almost all patients indicated that the social and spiritual questions need introspection for personal meaning, which makes thinking about these items relatively time-consuming when compared to physical symptoms. Some patients thought the questions touched upon topics that are sensitive. Going into dialogue about them did not fit in with their personal habits. However, this did not mean that these items were more difficult than the physical and psychological items, as some patients stated was stressed by HCPs. As one patient said: ‘The questions themselves are not that difficult. It is the thought process it triggers that makes them complex’ (PN10: female, 72). Another patient stated: ‘I thought the last questions [the social and spiritual items, TL] were harder. They concern things you cannot see on the outside. You must think about them: “How about that?”’ (AN03: female, 75). Patients also stated that the social and spiritual items prompted them to think about their life and situation and left them desiring more dialogue with others, whether their relatives or HCPs. A patient said: ‘But, most of all, it is nice that there are questions that you would not have thought of yourself. Questions that were not on the top of your mind, but that do matter. . . . And then the USD-4D helps you remember what you sometimes forget’ (AN05: female, 79). For some patients, responding to the social and spiritual questions was easier than responding to the physical and/or psychological items. One patient stated that HCPs told him the social and spiritual items would be harder to deal with than the others, which did not correspond with his experience: ‘Healthcare professionals keep telling me the social and spiritual questions are more difficult than the other questions. For me it’s just the other way around’ (AN11: male, 44). This shows it is important for HCPs to not project their uncomfortable feelings about the social and spiritual items on patients.

##### Trusting relationship

Patients expressed the importance of having a trusting relationship with HCPs. Discussing personal subjects, as addressed by the USD-4D items, both asks for and builds a proper patient-HCP relationship. One patient expressed: ‘You must be able to look each other in the eye. Sometimes you don’t know someone at all. That doesn’t matter, as long as the trust is there’ (PN01: male, 71)

Patients experiencing attunement felt more at ease using the USD-4D and going into dialogue about underlying needs to arrive at personalized care. One patient stated: ‘Healthcare professionals see me for who I am and how I feel. . . . I feel like myself. Feeling like myself helps me work with the nurses and doctors’ (PN03: female, 67). As one patient clearly stated, merely seeing a question on paper or in a digital app is not enough. You need to get a sense of what the question triggers and, in order for that to happen, you need to be able to discuss it with an HCP (AN04: female, 67). Furthermore, patients expressed a need for space to think about the questions: ‘At first glance you think, what does the item mean? But when you think about it, thoughts come to you on their own’ (PN11: male, 62).

## Discussion

This study identified what patients suffering from a life-limiting illness need when using the USD-4D to enable the identification of their unique needs to inform tailored care planning and provision. These needs were both internally and externally motivated and can be summarized in three themes: (1) feeling invited, (2) being aware of the purpose and function of the USD-4D, and (3) experiencing a personal and nonjudgmental approach.

Interestingly, AN and PN did not differ on a thematic level. Of course, AN were expressed more precisely and reflected patients’ experiences with the USD-4D, whereas PN reflected patients’ values. Both PN and AN should be taken into account when implementing and using any PROM, in this case the USD-4D, since these needs can develop over time.

In literature, what patients need to use a PROM is often synonymous with whether patients can technically complete PROMs.^24^ Hence, the emphasis is usually on the technical aspects of using PROMs rather than personal and emotional facets of answering the questions. This study showed that although a PROM might be valid and relevant; this is not an assurance of patients wanting to use it.

For all patients, being invited to use the USD-4D was the essential motivator. Only one other study has highlighted the importance of inviting patients to use a PROM.^46^ None of them experienced the initial invitation to use the PROM as burdensome. Patients wanted to decide for themselves whether to use the USD-4. Inviting patients leads to a more benevolent attitude toward using the instrument: patients did not feel pressured, did not feel compromised, and did not feel that it had to be done quickly. Patients stated that they did not feel burdened by being invited to use the USD-4D and that they had a sense of competent self-efficacy when it came to letting HCPs know if they did not want to use it.^14^

For patients to be willing to use the USD-4D more than once, it was important for them to experience the added value of using the PROM regularly and its impact on care and treatment. Patients stated that a lack of understanding and a lack of feedback from HCPs on their outcomes as well as perceiving the USD-4D was a checklist or only being used for research purposes discouraged them from using it. HCPs should always be attuned to patients and provide the information needed about the use of the USD-4D and the intention to apply the questions answered in a more personalized approach of care.

Using PROMs does not come without challenges. It is complex to gain any insight into how patients feel based solely on PROM outcome scores.^47^ Patients need to make judgments about their own personal situation and feelings that are subjective in nature and cannot be measured in terms of ‘hard’ functional outcomes. Furthermore, these subjective outcomes can be influenced by internal factors such as mood, expectations, time and sentiment, and external factors such as healthcare context and patient-HCP relation.^48^ This study shows that patients clearly express these personal and emotional preconditional needs by using the USD-4D to enhance their personal autonomy.^49,50^

Literature shows that the use of PROMs improves clinical care and enhances patients’ autonomy.^6 [Bibr bibr23-26323524241260426]^ In one study, however, general practitioners clearly dispute the benefit of using PROMs that capture multiple domains or dimensions, such as the USD-4D.^51^ Multidimensional PROMs could suffer from patients not answering all items due to their extensive lengths or HCPs focusing on certain items they prefer or with which they are more or less familiar. Participants from this study, however, expressed they did not perceive the USD-4D to be demanding to complete because it only contains a small number of items. This meets the preferences of general practitioners who preferred PROMs with fewer items that do not take up much time.^52^ When a PROM is a preferred choice of HCPs, this positively contributes to how the instrument becomes embedded in daily palliative care. As such, the structural use of the USD-4D in palliative care will facilitate patient-HCP dialogue, which improves personalized care.

### Strengths and limitations

A strength of this study is that the interviews capture the experiences of real-life day-to-day care in a hospice, home, or hospital setting for patients with a life expectancy of less than 1 year. Both patients that were familiar and unfamiliar with the USD-4D were included in this study and reflected on their experience with the PROM. Preliminary results were discussed within the research team and were discussed iteratively with a group of patients’ representatives (*n* = 2) and nurses (*n* = 2), enabling us to check whether statements were interpreted correctly and if anything was missed in the interviews. Finally, this study was conducted within an interdisciplinary team, ensuring that the results were analyzed from different perspectives.

Some limitations concerning the maximum variation in this study must be considered. For this first study of patients’ needs when using the USD-4D, for methodological reasons we limited our inclusion to patients sharing a Dutch cultural background.^53^ The reason for this is that the USD-4D questions on the social and spiritual dimension have not been validated for patients and families with a migrant background. Research has proven that communication with patients with a migration background demands intercultural competencies such as the awareness of cultures with high-context and low-context communication.^54,55^ Therefore, including representatives of other cultural backgrounds in this research would not be methodologically sound. First steps toward an adaption of the USD-4D questions for Dutch immigrant populations have been taken, which has resulted in conversation guides for entering into a dialogue with Dutch patients and families with a migration background.^55,56^

Finally, although key variations were identified and purposeful sampling was utilized, we did not succeed in achieving maximum variation on all of these variables. A recent study has shown that patients with a higher interest in participating in medical research are more likely to be female, more educated or have a higher health literacy.^54^ When considering a patient-centered approach in healthcare, low-educated patients tend to prefer a more directive, biomedical approach^57^ and are more likely to take a passive role.^58^ Overall, low-educated patients regard patient-centered care as less important.^59^ This might explain our difficulties in including this segment of the patient population.

On a thematic level, patients’ needs are not based on their educational level. However, AN might differ. When patients want to be invited to use the USD-4D, the way in which HCPs should do this can differ from patient to patient. Communication should therefore be adapted to the individual patient.

### Recommendations for clinical practice

A first recommendation concerns the connection between instrument and patient. Adequate use of PROMs in day-to-day palliative care is unique to every patient and, as such, individualized communication and building care relations are essential.^60,61^ HCPs are responsible for ensuring that patients’ preconditions are met.^62^ Patient-HCP communication that has been adequately adapted to the individual patient enables the patient to respond in a fitting manner. In line with the Netherlands Quality Framework for Palliative Care, patients need to be supported by an HCP in deciding whether or not to use a PROM. Furthermore, HCPs are tasked with enabling patients’ introspection by giving them room for their individual ways and approaching them accordingly.

A second recommendation concerns respecting and supporting patients’ self-efficacy. HCPs often perceive PROMs to be burdensome for patients and project this on to the patients. HCPs tend to decide for patients whether they are capable and willing to use the USD-4D. In so doing, HCPs act as a gatekeeper, making decisions for patients *a priori*.^63^ They ignore the fact that patients indicate they do not perceive being invited to use the USD-4D, or even a PROM in general, as burdensome and, more importantly, they want to be invited, which is something that honors their autonomy. The identified themes in this study emphasize the patients’ desire to make autonomous decisions.^9,10,14^ HCPs should therefore simply invite patients to use the USD-4D as part of setting up personal care. Furthermore, by putting patients at the helm of their own care needs, using the USD-4D, in turn, facilitates patients’ autonomy.^6^ Not being invited to use the USD-4D or HCPs preventing patients from getting access to it, that is, gatekeeping, threatens patients’ self-government.

Lastly, patients’ and HCPs’ needs should be equally leading in implementation strategies. Most studies tend to put emphasis on HCPs’ needs, pushing patients’ needs to the margins. Patients’ needs that are identified in this study can also inform the development of implementation strategies.^10^ Implementation strategies should focus not so much on patients’ behavior but be aimed at increasing HCPs’ skills in meeting patients’ needs: introducing and using PROMs, increasing their knowledge of the social and spiritual dimensions, and increasing their skills to properly attune with patients so they are able to support patients in using a PROM like the USD-4D autonomously.^64,65^

### Recommendations for future research

This is a first explorative study on what patients need to use the USD-4D in clinical care. Future research could look, first, at how the USD-4D can be optimally implemented in clinical practice and what strategies are most effective. To this end, it is essential to also look at what HCPs need to use the instrument in clinical care. Therefore, a separate study could look at the needs of HCPs when using the USD-4D.

Second, future research should focus on further developing practical methods for using the USD-4D in the diversity of clinical practice and transmural interprofessional collaboration. Moreover, research should focus on how PROMs can be naturally implemented in palliative care with the possibility of continuously learning from data as well as secondary use of this date in research studies to improve care.

Third, future research should focus on the preconditions for a confidential and open atmosphere to use the USD-4D and discuss the questions and issues it raises. This also requires studying the applicability of the USD-4D for patients with different cultural backgrounds or patients with a low(er) educational level.

Lastly, research should focus on patients in different locations with other diagnoses and in different stages of palliative care since they might have different needs when using the USD-4D.

## Conclusion

This study identified what patients need when using the USD-4D in clinical palliative care. Patients expressed that it is essential that they feel invited to use the USD-4D and feel autonomous in deciding to use the USD-4D and enter into dialogue. Moreover, patients want to be assured that completion will contribute to their clinical care delivery. Patients expressed that they need HCPs to be attuned to their personality when communicating the purpose and function of the USD-4D and in the dialogue between HCP and patients. As such, HCPs are tasked with setting the right preconditions for patients to use the USD-4D as intended.

## Supplemental Material

sj-docx-1-pcr-10.1177_26323524241260426 – Supplemental material for ‘It is important to feel invited’: what patients require when using the Utrecht Symptom Diary – 4 Dimensional, a qualitative exploration Supplemental material, sj-docx-1-pcr-10.1177_26323524241260426 for ‘It is important to feel invited’: what patients require when using the Utrecht Symptom Diary – 4 Dimensional, a qualitative exploration by Tom Lormans, Everlien de Graaf, Sita de Vries, Carlo Leget and Saskia Teunissen in Palliative Care and Social Practice

sj-docx-2-pcr-10.1177_26323524241260426 – Supplemental material for ‘It is important to feel invited’: what patients require when using the Utrecht Symptom Diary – 4 Dimensional, a qualitative exploration Supplemental material, sj-docx-2-pcr-10.1177_26323524241260426 for ‘It is important to feel invited’: what patients require when using the Utrecht Symptom Diary – 4 Dimensional, a qualitative exploration by Tom Lormans, Everlien de Graaf, Sita de Vries, Carlo Leget and Saskia Teunissen in Palliative Care and Social Practice

sj-docx-3-pcr-10.1177_26323524241260426 – Supplemental material for ‘It is important to feel invited’: what patients require when using the Utrecht Symptom Diary – 4 Dimensional, a qualitative exploration Supplemental material, sj-docx-3-pcr-10.1177_26323524241260426 for ‘It is important to feel invited’: what patients require when using the Utrecht Symptom Diary – 4 Dimensional, a qualitative exploration by Tom Lormans, Everlien de Graaf, Sita de Vries, Carlo Leget and Saskia Teunissen in Palliative Care and Social Practice

## Declarations

**Ethics approval and consent to participate:** The study was conducted in compliance with the principles of Good Clinical Practice and in accordance with the principles of the Declaration of Helsinki and the General Data Protection Regulation. As a part of the INZICHT project, The Medical Research Ethics Committee Utrecht confirmed that the Medical Research Involving Human Subjects Act did not apply to this study (19-602/C, 2019). All participants have been adequately informed about the study they were to be participating in prior to giving consent. Consent was either obtained in writing or verbally when written verbal consent could not be obtained.

**Consent for publication:** With consenting to participate to this research, all participants consented to the publication of the results of this research.

**Availability of data and materials:** Primary data used in this research can be acquired by contacting the second author of this paper.

## References

[1] Teunissen S. In palliative care symptoms mean everything. Symptoms & symptom management in palliative care for cancer patients. Utrecht: Utrecht University, 2007.

[2] World Health Organization. Palliative care, https://www.who.int/health-topics/palliative-care (2022, accessed 2 November 2022).

[3] IKNL/Palliactief. Netherlands Quality Framework for Palliative Care, https://palliaweb.nl/getmedia/f553d851-c680-4782-aac2-2520632f2e8d/netherlands-quality-framework-for-palliative-care_2.pdf (2017).

[4] Kyte DG Calvert M van der Wees PJ , et al. An introduction to patient-reported outcome measures (PROMs) in physiotherapy. Physiotherapy 2015; 101: 119–125. 25620440 10.1016/j.physio.2014.11.003

[5] Santana M-J Feeny D. Framework to assess the effects of using patient-reported outcome measures in chronic care management. Qual Life Res 2014; 23: 1505–1513. 24318085 10.1007/s11136-013-0596-1

[6] Eriksen J Bygholm A Bertelsen P. The association between patient-reported outcomes (PROs) and patient participation in chronic care: a scoping review. Patient Educ Couns 2022; 105: 1852–1864. 35090802 10.1016/j.pec.2022.01.008

[7] Hui D Bruera E. The Edmonton Symptom Assessment System 25 years later: past, present, and future developments. J Pain Symptom Manage 2017; 53: 630–643. 28042071 10.1016/j.jpainsymman.2016.10.370 PMC5337174

[8] Bruera E Kuehn N Miller MJ , et al. The Edmonton Symptom Assessment System (ESAS): a simple method for the assessment of palliative care patients. J Palliat Care 1991; 7: 6–9. 1714502

[9] Van der Baan FH Koldenhof JJ Nijs EJ , et al. Validation of the Dutch version of the Edmonton Symptom Assessment System. Cancer Med 2020; 9: 6111–6121. 32643871 10.1002/cam4.3253 PMC7476846

[10] IJzerman-Korevaar M de Graeff A Heijckmann S , et al. Use of a symptom diary on oncology wards. Cancer Nurs 2021; 44: E209–E220. 10.1097/NCC.0000000000000792 31990694

[11] Leget C. Art of living, art of dying: spiritual care for a good death. London/Philadelphia, PA: Jessica Kingsley Publishers, 2017.

[12] Haufe M Leget C Glasner T , et al. Spiritual conversation model for patients and loved ones in palliative care: a validation study. BMJ Support Palliat Care. Epub ahead of print June 2022. DOI: 10.1136/bmjspcare-2022-003569. 35710709

[13] Vermandere M Warmenhoven F van Severen E , et al. The Ars Moriendi model for spiritual assessment: a mixed-methods evaluation. Oncol Nurs Forum 2015; 42: 294–301. 10.1188/15.ONF.294-301 26148311

[14] de Vries S Lormans T de Graaf E , et al. The content validity of the items related to the social and spiritual dimensions of the Utrecht Symptom Diary – 4 Dimensional from a patient’s perspective: a qualitative study. J Pain Symptom Manage 2021; 61: 287–294.e2. 10.1016/j.jpainsymman.2020.07.036 32777457

[15] de Graaf E van Klinken M Zweers D , et al. From concept to practice, is multidimensional care the leading principle in hospice care? An exploratory mixed method study. BMJ Support Palliat Care 2020; 10: e5. 10.1136/bmjspcare-2016-001200 28167657

[16] Lormans T de Graaf E van de Geer J , et al. Toward a socio-spiritual approach? A mixed-methods systematic review on the social and spiritual needs of patients in the palliative phase of their illness. Palliat Med 2021; 35: 1071–1098. 33876676 10.1177/02692163211010384 PMC8189005

[17] Bradshaw A Santarelli M Mulderrig M , et al. Implementing person-centred outcome measures in palliative care: an exploratory qualitative study using Normalisation Process Theory to understand processes and context. Palliat Med 2021; 35: 397–407. 33249996 10.1177/0269216320972049 PMC7897789

[18] Müller E Mayer-Steinacker R Gencer D , et al. Feasibility, use and benefits of patient-reported outcome measures in palliative care units: a multicentre observational study. BMC Palliat Care 2023; 22: 6. 36641450 10.1186/s12904-022-01123-y PMC9839955

[19] Radionova N Becker G Mayer-Steinacker R , et al. The views of physicians and nurses on the potentials of an electronic assessment system for recognizing the needs of patients in palliative care. BMC Palliat Care 2020; 19: 45. 32247316 10.1186/s12904-020-00554-9 PMC7129326

[20] Krawczyk M Sawatzky R Schick-Makaroff K , et al. Micro-meso-macro practice tensions in using patient-reported outcome and experience measures in hospital palliative care. Qual Health Res 2019; 29: 510–521. 29542400 10.1177/1049732318761366

[21] Aiyegbusi OL Isa F Kyte D , et al. Patient and clinician opinions of patient reported outcome measures (PROMs) in the management of patients with rare diseases: a qualitative study. Health Qual Life Outcomes 2020; 18: 177. 32522194 10.1186/s12955-020-01438-5 PMC7288678

[22] van Muilekom MM Teela L van Oers HA , et al. Patients’ and parents’ perspective on the implementation of Patient Reported Outcome Measures in pediatric clinical practice using the KLIK PROM portal. Qual Life Res 2022; 31: 241–254. 34324137 10.1007/s11136-021-02950-x PMC8800898

[bibr23-26323524241260426] Wiering B de Boer D Delnoij D. Patient involvement in the development of patient-reported outcome measures: a scoping review. Health Expect 2017; 20: 11–23. 26889874 10.1111/hex.12442 PMC5217930

[24] Lapin BR Honomichl R Thompson N , et al. Patient-reported experience with patient-reported outcome measures in adult patients seen in rheumatology clinics. Qual Life Res 2021; 30: 1073–1082. 33170400 10.1007/s11136-020-02692-2

[25] Thestrup Hansen S Kjerholt M Friis Christensen S , et al. ‘I am sure that they use my PROM data for something important’. A qualitative study about patients’ experiences from a hematologic outpatient clinic. Cancer Nurs 2020; 43: E273–E282. 10.1097/NCC.0000000000000738 31361675

[26] Field J Holmes MM Newell D. PROMs data: can it be used to make decisions for individual patients? A narrative review. Patient Relat Outcome Meas 2019; 10: 233–241. 31534379 10.2147/PROM.S156291 PMC6681163

[27] Kahlke RM. Generic qualitative approaches: pitfalls and benefits of methodological mixology. Int J Qual Methods 2014; 13: 37–52.

[28] O’Brien BC Harris IB Beckman TJ , et al. Standards for reporting qualitative research. Acad Med 2014; 89: 1245–1251. 24979285 10.1097/ACM.0000000000000388

[29] Mitchell KR Brassil KJ Rodriguez SA , et al. Operationalizing patient-centered cancer care: a systematic review and synthesis of the qualitative literature on cancer patients’ needs, values, and preferences. Psychooncology 2020; 29: 1723–1733. 32715542 10.1002/pon.5500 PMC7901502

[30] Downar J Goldman R Pinto R , et al. The ‘surprise question’ for predicting death in seriously ill patients: a systematic review and meta-analysis. Can Med Assoc J 2017; 189: E484–E493. 10.1503/cmaj.160775 PMC5378508 28385893

[31] Long CO. Cultural and spiritual considerations in palliative care. J Pediatr Hematol Oncol 2011; 33:S96–S101. 10.1097/MPH.0b013e318230daf3 21952581

[32] Speck P. Culture and spirituality: essential components of palliative care. Postgrad Med J 2016; 92: 341–345. 26933233 10.1136/postgradmedj-2015-133369

[33] Creswell J. Qualitative inquiry and research design: choosing among five approaches. 3rd ed. London: SAGE Publications, 2013.

[34] Forero R Nahidi S de Costa J , et al. Application of four-dimension criteria to assess rigour of qualitative research in emergency medicine. BMC Health Serv Res 2018; 18: 120. 29454350 10.1186/s12913-018-2915-2 PMC5816375

[35] Heaton J. Reworking qualitative data. London: SAGE Publications, 2004.

[36] Thorne S. Secondary analysis in qualitative research: issues and implications. In: Morse J (ed.) Critical issues in qualitative research methods. London: SAGE Publications, 1994, pp. 263–279.

[37] Hennink MM Kaiser BN Marconi VC. Code saturation versus meaning saturation. Qual Health Res 2017; 27: 591–608. 27670770 10.1177/1049732316665344 PMC9359070

[38] Hennink M Kaiser BN. Sample sizes for saturation in qualitative research: a systematic review of empirical tests. Soc Sci Med 2022; 292: 114523. 34785096 10.1016/j.socscimed.2021.114523

[39] Braun V Clarke V. What can ‘thematic analysis’ offer health and wellbeing researchers? Int J Qual Stud Health Well-being 2014; 9: 26152. 25326092 10.3402/qhw.v9.26152 PMC4201665

[40] QSR International Pty Ltd. NVivo Qualitative Data Analysis Software.

[41] Knol ASL Huiskes M Koole T , et al. Reformulating and mirroring in psychotherapy: a conversation analytic perspective. Front Psychol 2020; 11: 318. Epub ahead of print 5 March 2020. DOI: 10.3389/fpsyg.2020.00318. 32194480 PMC7066200

[42] Phillippi J Lauderdale J. A guide to field notes for qualitative research: context and conversation. Qual Health Res 2018; 28: 381–388. 29298584 10.1177/1049732317697102

[43] World Medical Association. World Medical Association Declaration of Helsinki: ethical principles for medical research involving human subjects. JAMA 2013; 310: 2191. 24141714 10.1001/jama.2013.281053

[44] Shabani M Borry P. Rules for processing genetic data for research purposes in view of the new EU General Data Protection Regulation. Eur J Hum Genet 2018; 26: 149–156. 29187736 10.1038/s41431-017-0045-7 PMC5838983

[45] Central Government The Netherlands. Medical research involving human subjects act, https://wetten.overheid.nl/BWBR0009408/2020-01-01 (2020, last accessed 14 June 2024).

[46] Simon J Porterfield P Bouchal SR , et al. ‘Not yet’ and ‘Just ask’: barriers and facilitators to advance care planning – a qualitative descriptive study of the perspectives of seriously ill, older patients and their families. BMJ Support Palliat Care 2015; 5: 54–62. 10.1136/bmjspcare-2013-000487 24644192

[47] Willik EM Terwee CB Bos WJW , et al. Patient-reported outcome measures (PROMs): making sense of individual PROM scores and changes in PROM scores over time. Nephrology 2021; 26: 391–399. 33325638 10.1111/nep.13843 PMC8048666

[48] Hartog ID Willems DL van den Hout WB , et al. Influence of response shift and disposition on patient-reported outcomes may lead to suboptimal medical decisions: a medical ethics perspective. BMC Med Ethics 2019; 20: 61. 31510994 10.1186/s12910-019-0397-3 PMC6737596

[49] Mejdahl CT Schougaard LMV Hjollund NH , et al. PRO-based follow-up as a means of self-management support – an interpretive description of the patient perspective. J Patient Rep Outcomes 2018; 2: 38. 30238083 10.1186/s41687-018-0067-0 PMC6125260

[50] Kotronoulas G Papadopoulou C Simpson MF , et al. Using patient-reported outcome measures to deliver enhanced supportive care to people with lung cancer: feasibility and acceptability of a nurse-led consultation model. Support Care Cancer 2018; 26: 3729–3737. 29779057 10.1007/s00520-018-4234-x

[51] Litchfield I Greenfield S Turner GM , et al. Implementing PROMs in routine clinical care: a qualitative exploration of GP perspectives. BJGP Open 2021; 5: bjgpopen20X101135. 10.3399/bjgpopen20X101135 PMC7960526 33199306

[52] Turner GM Litchfield I Finnikin S , et al. General practitioners’ views on use of patient reported outcome measures in primary care: a cross-sectional survey and qualitative study. BMC Fam Pract 2020; 21: 14. 31980021 10.1186/s12875-019-1077-6 PMC6979354

[53] Sousa VD Rojjanasrirat W. Translation, adaptation and validation of instruments or scales for use in cross-cultural health care research: a clear and user-friendly guideline. J Eval Clin Pract 2011; 17: 268–274. 20874835 10.1111/j.1365-2753.2010.01434.x

[54] Kripalani S Goggins K Couey C , et al. Disparities in research participation by level of health literacy. Mayo Clin Proc 2021; 96: 314–321. 33549253 10.1016/j.mayocp.2020.06.058 PMC7874435

[55] Leget C Teunissen S Haufe M , et al. In gesprek over levensvragen. Het Diamant-Model voor Nederlandse Moslims. Utrecht: University of Humanistic Studies, 2022.

[56] Leget C Teunissen S Haufe M , et al. In gesprek over levensvragen. Sranang Djamanti, Het diamant-model voor Surinaamse Nederlanders. Utrecht: University of Humanistic Studies, 2022.

[57] de Haes H . Dilemmas in patient centeredness and shared decision making: a case for vulnerability. Patient Educ Couns 2006; 62: 291–298. 16859860 10.1016/j.pec.2006.06.012

[58] Say R Murtagh M Thomson R. Patients’ preference for involvement in medical decision making: a narrative review. Patient Educ Couns 2006; 60: 102–114. 16442453 10.1016/j.pec.2005.02.003

[59] Rademakers J Delnoij D Nijman J , et al. Educational inequalities in patient-centred care: patients’ preferences and experiences. BMC Health Serv Res 2012; 12: 261. 22900589 10.1186/1472-6963-12-261 PMC3467160

[60] Carmona C Crutwell J Burnham M , et al. Shared decision-making: summary of NICE guidance. BMJ 2021; 373: n1430. 10.1136/bmj.n1430 34140279

[61] Matthys J. Shared decision making: an ideal and an art. BMJ 2021; 374: n2033. 10.1136/bmj.n2033 34407974

[62] Hupkens S Goumans M Derkx P , et al. Nurse’s attunement to patient’s meaning in life – a qualitative study of experiences of Dutch adults ageing in place. BMC Nurs 2020; 19: 41. 32477004 10.1186/s12912-020-00431-z PMC7236336

[63] Kars MC van Thiel GJ van der Graaf R , et al. A systematic review of reasons for gatekeeping in palliative care research. Palliat Med 2016; 30: 533–548. 26577927 10.1177/0269216315616759

[64] Alexander KE Brijnath B Mazza D. Barriers and enablers to delivery of the Healthy Kids Check: an analysis informed by the Theoretical Domains Framework and COM-B model. Implement Sci 2014; 9: 60. 24886520 10.1186/1748-5908-9-60 PMC4047437

[65] van Leeuwen LM Pronk M Merkus P , et al. Barriers to and enablers of the implementation of an ICF-based intake tool in clinical otology and audiology practice: a qualitative pre-implementation study. PLoS One 2018; 13: e0208797. 10.1371/journal.pone.0208797 PMC6289452 30533057

