# Supplementary material for: ‘It is important to feel invited’: what patients require when using the Utrecht Symptom Diary – 4 Dimensional, a qualitative exploration
Source: Palliat Care Soc Pract. 2024 Jun 20;18:26323524241260426. doi: 10.1177/26323524241260426 (PMC11191620; doi:10.1177/26323524241260426)
Supplement: sj-docx-2-pcr-10.1177_26323524241260426 – Supplemental material for ‘It is important to feel invited’: what patients require when using the Utrecht Symptom Diary – 4 Dimensional, a qualitative exploration [file sj-docx-2-pcr-10.1177_26323524241260426.docx]

**Utrecht Symptom Diary – 4 Dimensional**


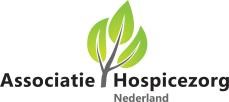

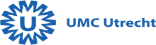


**The scores are provided by:** 0 patient 0 loved one/ relative 0 caregiver

**I have**

| No pain | 0 | 1 | 2 | 3 | 4 | 5 | 6 | 7 | 8 | 9 | 10 | Worst possible pain |
| --- | --- | --- | --- | --- | --- | --- | --- | --- | --- | --- | --- | --- |
| No sleeping problems | 0 | 1 | 2 | 3 | 4 | 5 | 6 | 7 | 8 | 9 | 10 | Worst possible sleeping problems |
| No dry mouth | 0 | 1 | 2 | 3 | 4 | 5 | 6 | 7 | 8 | 9 | 10 | Worst possible dry mouth |
| No dysphagia | 0 | 1 | 2 | 3 | 4 | 5 | 6 | 7 | 8 | 9 | 10 | Worst possible dysphagia |
| No lack of appetite | 0 | 1 | 2 | 3 | 4 | 5 | 6 | 7 | 8 | 9 | 10 | Worst possible lack of appetite |
| No consitpation | 0 | 1 | 2 | 3 | 4 | 5 | 6 | 7 | 8 | 9 | 10 | Worst possible constipation |
| *Other* |  |  |  |  |  |  |  |  |  |  |  |  |
| ………………………………. | 0 | 1 | 2 | 3 | 4 | 5 | 6 | 7 | 8 | 9 | 10 |  |
| **I feel** |  |  |  |  |  |  |  |  |  |  |  |  |
| No nausea | 0 | 1 | 2 | 3 | 4 | 5 | 6 | 7 | 8 | 9 | 10 | Worst possible nausea |
| No shortness of breath | 0 | 1 | 2 | 3 | 4 | 5 | 6 | 7 | 8 | 9 | 10 | Worst possible shortness of breath |
| No fatigue | 0 | 1 | 2 | 3 | 4 | 5 | 6 | 7 | 8 | 9 | 10 | Worst possible fatigue |
| Not different than usual | 0 | 1 | 2 | 3 | 4 | 5 | 6 | 7 | 8 | 9 | 10 | Very different than usual |
| Not anxiety | 0 | 1 | 2 | 3 | 4 | 5 | 6 | 7 | 8 | 9 | 10 | Worst possible anxiety |
| No depressed mood | 0 | 1 | 2 | 3 | 4 | 5 | 6 | 7 | 8 | 9 | 10 | Worst possible depressed mood |
| *Other* |  |  |  |  |  |  |  |  |  |  |  |  |
| ………………………………. | 0 | 1 | 2 | 3 | 4 | 5 | 6 | 7 | 8 | 9 | 10 |  |
| **At this moment, I experience** |  |  |  |  |  |  |  |  |  |  |  |  |
| Best possible well-being | 0 | 1 | 2 | 3 | 4 | 5 | 6 | 7 | 8 | 9 | 10 | Worst possible well-being |
| **At this moment, my life is** | |  |  |  |  |  |  |  |  |  |  |  |
| Worthwhile 0 | | 1 | 2 | 3 | 4 | 5 | 6 | 7 | 8 | 9 | 10 | Not worthwhile |
|  |  |  |  |  |  |  |  |  |  |  |  |  |
| **According to you, which symptoms have to be prioritized?**  1.  2. | | | | | | | | | | | | |

**The scores are provided by:** 0 patient 0 loved one/ relative 0 caregiver

**I take time for myself**

| yes | 0 | 1 | 2 | 3 | 4 | 5 | 6 | 7 | 8 | 9 | 10 | Not at all |
| --- | --- | --- | --- | --- | --- | --- | --- | --- | --- | --- | --- | --- |
| **I can bear what happens to me** |  |  |  |  |  |  |  |  |  |  |  |  |
| yes | 0 | 1 | 2 | 3 | 4 | 5 | 6 | 7 | 8 | 9 | 10 | Not at all |
| **I can let my loved ones go** |  |  |  |  |  |  |  |  |  |  |  |  |
| yes | 0 | 1 | 2 | 3 | 4 | 5 | 6 | 7 | 8 | 9 | 10 | Not at all |
| **I feel a sense of balance in my life** |  |  |  |  |  |  |  |  |  |  |  |  |
| yes | 0 | 1 | 2 | 3 | 4 | 5 | 6 | 7 | 8 | 9 | 10 | Not at all |
| **My thoughts about the end of life give me peace of mind** |  |  |  |  |  |  |  |  |  |  |  |  |
| yes | 0 | 1 | 2 | 3 | 4 | 5 | 6 | 7 | 8 | 9 | 10 | Not at all |

**Furthermore, I would like to let you know…..**

1.

2.

© USD-4D EPZ Utrecht English version 1.0
