# Supplementary material for: ‘It is important to feel invited’: what patients require when using the Utrecht Symptom Diary – 4 Dimensional, a qualitative exploration
Source: Palliat Care Soc Pract. 2024 Jun 20;18:26323524241260426. doi: 10.1177/26323524241260426 (PMC11191620; doi:10.1177/26323524241260426)
Supplement: sj-docx-3-pcr-10.1177_26323524241260426 – Supplemental material for ‘It is important to feel invited’: what patients require when using the Utrecht Symptom Diary – 4 Dimensional, a qualitative exploration [file sj-docx-3-pcr-10.1177_26323524241260426.docx]

**Appendix 2. Topic list**

| **Before commencement of interview** | |
| --- | --- |
|  | |
| **Rapport building** | How do you feel at the moment? |
|  |  |
|  |  |
| **Commencement of interview, recorder is running** | |
|  | |
| **Utrecht Symptom Diary – 4 Dimensional** | Do you recall using the USD-4D? |
|  |  |
|  |  |
| **Patients’ needs** | |
|  | |
| **Patient’s self** | When is the best moment for you to use the USD-4D? |
|  | What emotional state is preferable for you when using the USD-4D? |
|  | Do you know to what end the USD-4D is used? |
|  |  |
| **The other** | How do you like to be approached by the HCP? |
|  | What attitude motivates you to use the USD-4D? |
|  | Do HCPs invite you to use the USD-4D? |
|  |  |
| **Location** | Where do you like to use the USD-4D best? |
|  | How does privacy affect using the USD-4D? |
|  |  |
| **Time** | How much time does it cost you to use the USD-4D? |
|  | Does using the USD-4D burden you? |
|  |  |
| **Wrap-up** | |
|  | |
| **Final question** | Is there anything you would like to add? |
